# Metabolomic and Transcriptomic Analyses Reveal the Effects of Grafting on Nutritional Properties in Eggplant

**DOI:** 10.3390/foods12163082

**Published:** 2023-08-17

**Authors:** Yaqin Yan, Wuhong Wang, Tianhua Hu, Haijiao Hu, Jinglei Wang, Qingzhen Wei, Chonglai Bao

**Affiliations:** Institute of Vegetable, Zhejiang Academy of Agricultural Science, Hangzhou 310021, China; zkyyanyaqinl@163.com (Y.Y.); wangwh@zaas.ac.cn (W.W.); huth@mail.zaas.ac.cn (T.H.); huhj@mail.zaas.ac.cn (H.H.); wangjingle@mail.zaas.ac.cn (J.W.); weiqz@mail.zaas.ac.cn (Q.W.)

**Keywords:** grafted eggplant, rootstocks, metabolome, transcriptome, fruit quality

## Abstract

Grafting has a significant impact on the botany properties, commercial character, disease resistance, and productivity of eggplants. However, the mechanism of phenotypic modulation on grafted eggplants is rarely reported. In this study, a widely cultivated eggplant (*Solanum. melongena* cv. ‘Zheqie No.10’) was selected as the scion and grafted, respectively, onto four rootstocks of TOR (*S. torvum*), Sa (*S. aculeatissimum*), SS (*S. sisymbriifolium*), and Sm64R (*S. melongena* cv. ‘Qiezhen No. 64R’) for phenotypic screening. Physiological and biochemical analysis showed the rootstock Sm64R could improve the fruit quality with the increasing of fruit size, yield, and the contents of total soluble solid, phenolic acid, total amino acid, total sugar, and vitamin C. To further investigate the improvement of fruit quality on Sm64R, a transcriptome and a metabolome between the Sm64R-grafted eggplant and self-grafted eggplant were performed. Significant differences in metabolites, such as phenolic acids, lipids, nucleotides and derivatives, alkaloids, terpenoids, and amino acids, were observed. Differential metabolites and differentially expressed genes were found to be abundant in three core pathways of nutritional qualities, including biosynthesis of phenylpropanoids, phospholipids, and nucleotide metabolism. Thus, this study may provide a novel insight into the effects of grafting on the fruit quality in eggplant.

## 1. Introduction

Eggplant (*Solanum melongena* L.) is a member of the *Solanaceae* family and genus Solanaceous, an economically essential vegetable cultivated worldwide (Daunay, 2008). Eggplant has significant economic value, as it is rich in protein, unsaturated fatty acids, alkaloids, minerals, flavonoids, vitamins, dietary fibers, and antioxidants with a unique flavour and offers rich nutrition benefits to humans [1].

Grafting is a well-developed technique in vegetables, combining a scion and a tolerant rootstock to form a resistant plant with various traits [2]. Grafting technology can effectively control diseases and insect pests, improve fruit quality, increase cold tolerance, overcome continuous cropping damage obstacles, and enhance plant root nutrient absorption capacity [3]. Several studies have shown the significant impact of rootstock on fruit size, shape, flesh colour, texture, flavour, aroma, and sugar in grafted cultivars [4]. Many contradictory studies have been conducted to determine whether grafting improves or degrades fruit quality [5]. Higher yields and larger fruits were reported in eggplant, tomato, watermelon, and cucumber when plants were grafted [6,7,8,9]. Interspecific hybrid pumpkin rootstock grafting decreases watermelon fruits taste, total soluble solids (TSS), and sugar content [10]. According to several studies on tomatoes, watermelon, and other vegetables, the type of rootstock and scion in a grafted tree are related depending on various physiological factors [11]. Thus, the selection of the appropriate graft combination may be essential for yield efficiency, plant vigour, and fruit quality. Hence, even quality traits may differ depending on how grafting and cultivar interact.

Tolerant rootstocks are currently used for eggplant grafting to confer tolerance to various soil-borne diseases. The rootstocks used for eggplant grafting are the same species. A previous study reported that grafting the eggplant (scion) onto *S. torvum* (rootstock) may increase fruit size but reduce the lightness and saturation of their colour [12]. However, little evidence is known on the direct effects of rootstock on the scion physiology and nutritional quality of the eggplant. Techniques such as metabolomics, transcriptomics, and proteomics could help to elucidate the specific and underlying molecular mechanisms of grafting-dependent biochemical processes. Integrated metabolome and transcriptome analysis of the grafted cucumber fruit showed metabolites and genes involved in sugar metabolism and linoleic acid and amino acid biosynthesis [13]. In a metabolomic and transcriptomic analysis of pumpkin-grafted watermelon and ungrafted watermelon, the soluble solid content (SSC), total sugars, total amino acids, and total acids of the grafted watermelon were found to be drastically changed, including changes in the sugar metabolism, linoleic acid, and amino acid biosynthesis pathways [14].

In this study, an eggplant was grafted onto four rootstocks TOR (*S. torvum*), Sa (*S. aculeatissimum*), SS (*S. sisymbriifolium*), and Sm64R (*S. melongena* cv. ‘Qiezhen No. 64R’), and a self-grafted was used as the control. All these four rootstocks have been reported to confer tolerance to various soil-borne diseases; all are currently used for eggplant grafting worldwide. In order to select the appropriate graft combination to improve the fruit quality, various physiological and biochemical parameters were measured. The rootstock Sm64R increased the fruit size, total solid content, phenolic acid content, amino acid content, and vitamin C content, with no significant influence on the anthocyanin content. To explore the fruit quality of eggplant grafted onto Sm64R, an integrated metabolomic and transcriptomic analysis was performed to examine the correlation among different nutrition-related components from self-grafted plants and fruits from plants grafted onto Sm64R. Our findings may provide candidate metabolites or genes for the physiological mechanism study of fruit quality in grafted eggplant.

## 2. Materials and Methods

### 2.1. Experimental Materials and Sampling

Eggplant (*S. melongena* cv. ‘Zheqie No.10’) was grafted onto four rootstocks Sa (*S. aculeatissimum*), SS (*S. sisymbriifolium*), TOR (*S. torvum*), and Sm64R (*S. melongena* cv. ‘Qiezhen No. 64R’). All the rootstocks and sections were obtained from the Zhejiang Academy of Agricultural Science, Hangzhou, Zhejiang, China.

From March to July of 2022, the grafted seedlings were cultured in a greenhouse at the Qiaosi comprehensive test field of the Zhejiang Academy of Agricultural Sciences, Hangzhou, China (120.28° E, 30.36° N). The rows were 100 cm apart, and the plant-to-plant space was kept at 50 cm. This field has been planted with eggplant for the past three years. Randomized complete block design with three replications was used. Each plot has 60 plants in a single row. Self-grafted scion plants were used as the control (SG). Each grafting combination was performed with sixteen replicates. Three independent plants of each grafting combination were chosen randomly as biological replicates. The fruits of eggplant were harvested from the third branch of the plant. Maturity is determined by the connection between the eggplant fruit sepals and the fruit. All samples were taken from eggplant fruits with consistent maturity. For metabolome and transcriptome analysis, the exocarp in the middle part of eggplant fruit was excised into 2 cm × 2 cm cubes with peels, preserved immediately in liquid nitrogen, and stored at −80 °C.

### 2.2. Measurement of Physiological Traits of Eggplant Fruit

The methods for measuring the total phenolic content, total anthocyanin content, flavanol content, soluble sugar content, organic acid contents, soluble protein content, free amino acid contents, and vitamin C content were adapted from the method of Zhang et al. [15]. A handheld refractometer with the unit of % was applied to determine the TSS content in the mixed fruits. The total content of amino acids was determined using ninhydrin hydrate colorimetry [16]. The content of total soluble sugars was determined based on anthrone sulfuric acid [15]. The Folin–Ciocalteu method was used to determine the total phenolic content [17]. The total anthocyanin content was determined using the hydrochloric acid-methanol extraction method [18]. The p-DMACA method was used to determine the flavanol content [19]. The phenolic components were determined following the manipulation of Zhao et al. [20]. The vitamin C content was determined based on the Fe^3+^ reduction method [21]. Each sample was analysed in triplicate.

### 2.3. Widely Targeted Metabolite Identification and Quantification

The freeze-dried samples were crushed for 1.5 min at 30 Hz in a mixer mill (MM 400, Retsch, Haan, Germany) using zirconia beads. The powder (50 mg) was weighed out and dissolved in 1.2 mL of 70% methanol extract. After centrifugation at 12,000 rpm for 3 min, the supernatant was filtered via a microporous filter membrane (0.22 μm point size) before UPLC–MS/MS analysis. Then, the sample extracts were analysed using an ultra-performance liquid chromatography system (ExionLC AD) and tandem mass spectrometry system (Applied Biosystems 6500 QTRAP). A prior method was used to conduct a qualitative analysis of primary and secondary MS data [22]. Metabolite data were statistically analysed by MWDB database (MetWare Biological Science and Technology Co., Ltd., Wuhan, China) as well as publicly available metabolite databases. In metabolome analysis, the identified metabolites were used to perform principal component analysis (PCA) to show the correlations between the samples. The differentially accumulated metabolites (DAMs) between the samples were identified using orthogonal partial least squares discriminant analysis (OPLS-DA). In the analysis, the criteria log2(fold change) > 1 and variable significance in the project (VIP) ≥ 1 were used. The DAMs were mapped to the respective metabolic pathways using the Kyoto Encyclopedia of Genes and Genomes (KEGG) database. ClusterProfiler was then used to perform a KEGG enrichment analysis. The R environment (https://www.r-project.org/(accessed on 1 July 2022)) was used for all data analysis. Each sample was analysed in triplicate.

### 2.4. RNA Sequencing (RNA-seq) Analyses and Differentially Expressed Genes (DEGs)

Following the manufacturer’s instructions, total RNA was extracted from the freeze-dried samples, and sequencing libraries were generated with the NEBNext Ultra RNA Library Prep Kit for Illumina (New England Biolabs, Ipswich, MA, USA). The library preparations were sequenced using 150 bp paired-end reads on an Illumina HiSeq 2500 platform. Fastq format raw reads were filtered using custom PERL scripts to produce clean data. The reference genome of Solanum melongena (http://eggplant-hq.cn/Eggplant/home/index/ (accessed on 1 July 2022)) was aligned using paired-end clean reads. FPKM (Fragments per kilobase of transcript per million) was used to calculate the expression levels of the genes. Differentially expressed genes were defined as those with an adjusted log_2_fold change >1 or 1 and a *p* value < 0.05 in the DESeq2 output. KEGG enrichment analysis was performed using the clusterProfiler Bioconductor program. Each sample was analysed in triplicate.

## 3. Results

### 3.1. Phenotype and Physiological Traits of Eggplant Fruit from Plants Grafted on Different Rootstocks

*S. melongena* cv. ‘Zheqie No.10’, a widely cultivated purple eggplant in Zhejiang Province, was used as a section in this study. Zheqie No. 10 was grafted with four rootstock genotypes (SS, Sa, TOR, and Sm64R), and the self-grafted plant SG was used as a control (Table 1). The eggplant fruits of each scion rootstock combination were harvested at the maturation stage. Fruits from different rootstock showed significant morphological variations. The eggplant fruits grafted onto Sm64R had the maximum fruit weight and fruit length compared to the self-grafted control SG (Table 1).

To examine the impact of rootstocks on eggplant fruit quality, hardness, total soluble solids (TSS), and titratable acidity (TA) were measured (Table 1). The TSS is a critical quality indicator that represents the fruits’ sweetness and consists of sugars and acids with a small number of dissolved proteins, vitamins, phenolics, and minerals. The results showed that an apparently higher TSS (15.78°) was recorded for the eggplant fruits grafted onto Sm64R rootstock, and the fruits grafted onto SS rootstock exhibited the lowest TSS (13.38°). Higher fruit hardness was observed for the eggplant fruits obtained from plants grafted onto Sa rootstock, but statistically, there was no difference among the different grafting combinations. The content of titratable acid was significantly decreased in the Sm64R-grafted eggplant and TOR-grafted eggplant compared to self-grafted eggplant SG.

Compared with the self-grafted eggplant SG, grafting significantly decreased the contents of the flavonoids, anthocyanin, total sugar, and vitamin C in the Sa-grafted eggplant (Table 2). Compared with the self-grafted eggplant SG, a lower flavonoids content, anthocyanin content, and vitamin C were observed in SS-grafted eggplant, but there were not significant effects on the phenolic acid content and free amino acid (FAA) contents. Compared with the self-grafted eggplant SG, the TOR-grafted eggplant showed increased amounts of FAA and vitamin C, and decreased amounts of flavonoids content, anthocyanin content, phenolic acid content and total sugar content (Table 2). Higher content of phenolic acid content, FAA, total sugar, and vitamin C was observed for the eggplant fruits obtained from the Sm64R-grafted eggplant compared to the self-grafted plants SG. These physiological changes suggested that the rootstock Sm64R could improve the fruit quality, and the eggplant flavours were noticeably different between the Sm64R-grafted eggplant and the self-grafted eggplant SG.

### 3.2. Widely Targeted Metabolomic Analysis

**Figure 1:**
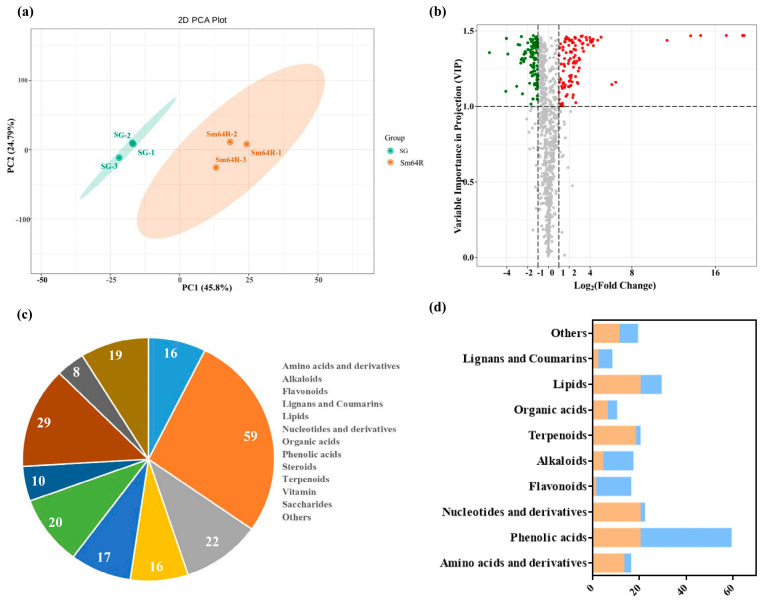
Metabolomic analyses between Sm64R-grafted eggplant and self-grafted eggplant SG. (**a**) Principal component analysis of the metabolites in Sm64R-grafted eggplant and self-grafted eggplant SG. (**b**) Volcano plot analysis of different abundances of metabolites. Red color indicates high abundance, gray color indicates middle value, and green color indicates low abundance. (**c**) The number of different abundances of metabolites in each class. (**d**) Statistics of the different abundances of metabolites in each class. Orange color represents the number of higher accumulated metabolites; blue color represents the number of lower accumulated metabolites.

A broad targeted liquid chromatography–mass spectrometry (LC-MS) method was used to comprehensively profile eggplant fruits after grafting with rootstock Sm64R. The PCA results showed that the first two principal components explained 45.8 and 24.79 of the data variance, respectively (Figure 1a). The variable importance in the projection (VIP) value was set as ≥1.0, and a *p*-value of ≤0.5 was used as the threshold for significant differences. A total of 216 different accumulated metabolites (DAMs) were detected in our study, including 59 phenolic acids, 29 lipids, 22 nucleotides and derivatives, 20 terpenoids, 17 alkaloids, 16 amino acids and derivatives, 16 flavonoids, 10 organic acids, 8 lignans and coumarins, 2 steroids, and 17 others (Figure 1b,c). The top six categories with the most significant number of significantly differentially accumulated metabolites identified from the Sm64R-grafted eggplant and self-grafted eggplant SG were phenolic acids, lipids, nucleotides and derivatives, terpenoids, alkaloids, and amino acids and derivatives. The Kyoto Encyclopedia of Genes and Genomes (KEGG) pathway enrichment analysis was applied to the DAMs to highlight their potential biological functions. KEGG enrichment analysis indicated that DAMs were mainly associated with biosynthesis of various plant secondary metabolites, nucleotide metabolism, pyrimidine metabolism, ABC transporters, biosynthesis of cofactors, and phenylalanine, tyrosine, and tryptophan biosynthesis (Figure S2).

### 3.3. Significantly Differently Accumulated Bioactive Compounds between Sm64R-Grafted Eggplant and Self-Grafted Eggplant SG

Eggplant has been shown to contain a wide range of bioactive compounds, such as phenolic compounds, alkaloids, terpenoids, vitamins, dietary fibre, and minerals. These compounds have a variety of biological activities, including antioxidant, antitumoral, antimicrobial, antiparasitic, insecticidal, and herbicidal activities [23].

Phenolic acids (PAs) are the most common bioactive compounds in plants and may function as reducing agents [24]. The most significant number of DAMs identified from the Sm64R-grafted eggplant and self-grafted eggplant SG were classified as PAs, which have been shown to play essential roles in human nutrition and influence the sensory characteristics of vegetable products [25]. A comparison of the self-grafted SG showed that 59 phenolic acids were differentially accumulated in the Sm64R-grafted eggplant. In contrast to the self-grafted control SG, the Sm64R-grafted eggplant identified 20 and 39 differentially accumulated metabolites that were upregulated and downregulated, respectively (Figure S1). The relative content profiles of the phenolic acids from the DAMs showed that 20 phenolic acid compounds, such as salicin, vanillic acid, and caffeoylquinic acid, had higher contents in the Sm64R-grafted eggplant than in the self-grafted eggplant SG (Table S1). In particular, the grevilloside F and salicin contents in Sm64R-grafted eggplant were 18.75 and 14.60 times those in self-grafted eggplant SG, respectively. These results indicated that phenolic acids were the primary differential metabolites between the Sm64R-grafted eggplant and self-grafted eggplant SG.

Alkaloids are the major biologically active compounds found in Solanaceae and play essential roles in the human diet and affect the sensory traits of vegetable products [26]. Many alkaloids display strong bioactivity, such as caffeine, atropine, and cocaine, whereas many other alkaloids, such as pyrrolizidine alkaloids, are harmful to humans. Four and thirteen up- and downregulated differentially accumulated alkaloids, respectively, were identified in the Sm64R-grafted eggplant compared to self-grafted eggplant SG (Figure 1c; Table S1). Steroid alkaloids are abundant among the members of the Solanaceae family. The most predominant steroid alkaloids in eggplant are solasonine and solamargine [27]. In this study, steroid alkaloids, including β2-solanine, solasodine, scopolamine, khasianine, γ-tomatine, and dehydrotomatine, were detected in all samples, which showed no significant difference between Sm64R-grafted eggplant and self-grafted eggplant SG. However, demissidine was present at higher levels in the Sm64R-grafted eggplant than in the self-grafted eggplant SG. In particular, the content of demissidine in Sm64R-grafted eggplant was 4.09 times that in self-grafted eggplant SG. Desmissidine exists in some Chinese herbal medicines (such as *S. nigrum*) and is used as its active ingredient. These compounds have antitumour, antibacterial, anti-inflammatory, and other pharmacological activities. In addition, the levels of most alkaloids were significantly lower in Sm64R than in self-grafted SG. The results indicated that the rootstock Sm64R could modulate the accumulation of distinct metabolites in alkaloid groups.

Terpenoids are the most abundant and structurally diverse natural products in many plants. In total, 20 differentially accumulated terpenoids were identified in eggplant fruits, including 11 triterpenes, 6 sesquiterpenoids, 2 triterpene saponins, and 1 diterpenoid. Eighteen of twenty DAMs were significantly higher in Sm64R than in self-grafted SG (Figure 1c; Table S1). The results showed that the contents of 3-O-p-coumaroyloleanolic acid, 2,3,23-trihydroxy-30-nor lean-12,20 (21)-dien-28-oic acid, 11,12-O-isopropyfidenesolajiangxin F, JiangxiBaiyingsu I, septemlobin D, norarjunolic acid, oleanolic acid-3-O-xylosyl-glucuronide, epinootkatol, and bubonic acid were higher in the Sm64R-grafted eggplant than in the self-grafted eggplant SG. The results indicate that rootstock Sm64R could significantly promote the accumulation of terpenoids in eggplant fruits.

### 3.4. Significantly Different Nitrogen Metabolism between Sm64R-Grafted Eggplant and Self-Grafted Eggplant SG

Nitrogen is integral to amino acid, chlorophyll, and nucleic acid synthesis [28]. Amino acids are key components of the human diet, playing important functions in dietary nutrition and flavour [29]. In contrast to the self-grafted eggplant SG, the Sm64R-grafted eggplant identified 13 and 3 differentially accumulated metabolites with higher and lower accumulation, respectively (Figure 1b). More amino acids, including Phe-Ile, S-ribosyl-L-homocysteine, L-leucyl-L-leucine, glycylphenylalanine, Glu-Phe, L-leucyl-L-phenylalanine, L-γ-glutamyl-L-leucine, N-acetyl-L-tyrosine, and L-phenylalanyl-L-phenylalanine, were detected at a higher level in Sm64R-grafted eggplant than in self-grafted eggplant SG (Table S1). Therefore, grafting may contribute to the accumulation of amino acids and their derivatives in eggplant fruits.

Nucleotides and their derivatives are nutritional compounds, and nucleotides have been identified as key flavor potentiators that promote food palatability [30]. Specifically, the rootstock Sm64R exhibited 20 and 2 up- and downregulated differentially accumulated metabolites, respectively. In particular, the levels of most nucleotides and derivatives were significantly higher in Sm64R-grafted eggplant than in self-grafted eggplant SG (Table S1).

Lipids are essential nutrients for humans and have a key role in flavor and taste in food (Uauy and Castillo, 2003). In total, 29 differentially accumulated lipids were identified in eggplant fruits, including 7 lysophosphatidylcholines (LysoPCs), 7 lysophosphatidylethanolamines (LysoPEs), 6 free fatty acids, 5 glycerol ester, and 1 phosphatidylcholine (PC) (Table S1). The SG vs. Sm64R comparison identified 20 and 9 up- and downregulated differentially accumulated metabolites, respectively (Figure 1c). Phospholipids are macromolecules that are vital in cells and perform physiological regulating activities. More phospholipids, including 6 LysoPCs and 8 LysoPEs, were found at a higher level in Sm64R-grafted eggplant than in self-grafted eggplant.

### 3.5. An Overview of the Transcriptomic Data

**Figure 2:**
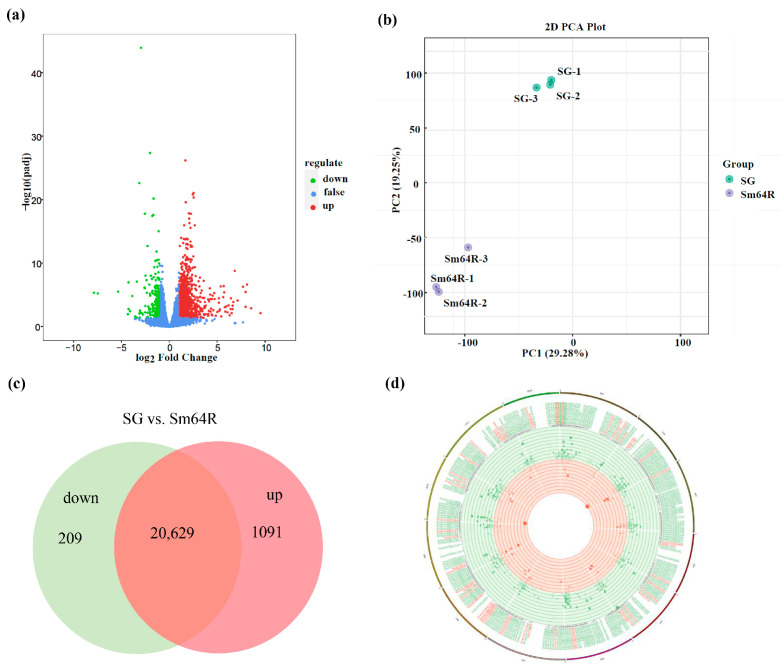
Transcriptomic analyses between Sm64R-grafted eggplant and self-grafted eggplant SG. SG. (**a**) Volcano plot analysis of DEGs. Red color shows high expression level, blue color shows middle value, and green color shows low expression value. (**b**) Principal component analysis of eggplant fruit samples. (**c**) Number of differentially expressed genes (DEGs) between Sm64R-grafted eggplant and self-grafted eggplant. (**d**) Circos plot analysis of the distribution of the DEGs along the physical map of the eggplant genome.

Transcriptome analysis was conducted to identify the gene expression profiles of the Sm64R-graft eggplant compared to the self-grafted control SG (Figure 2a). The clean reads have been submitted to SRA with the accession number PRJNA978363. A total of 59.75 Gb clean data were obtained from the RNA-seq dataset. Based on all clean reads, 33,684 coding genes were matched to the high-quality eggplant reference genome [31]. All of the mapped sequencing reads were used to analyse differentially expressed genes (DEGs) in DEseq2 with the criteria of FDR (false discovery rate) <0.05 and fold change ≥2. The PCA results showed that the first two principal components explained 29.28 and 19.25 of the data variance, respectively (Figure 2b). In total, 1300 genes were differentially expressed between SG and Sm64R samples, with 1091 up- and 208 downregulated genes, respectively (Figure 2c). Circos plots depicting the distribution of the DEGs along the physical map of the eggplant genome are shown in Figure 2d. The findings revealed that the DEG libraries contained a wide range of genes and that complex molecular regulatory pathways were involved in the formation of fruit quality.

**Figure 3:**
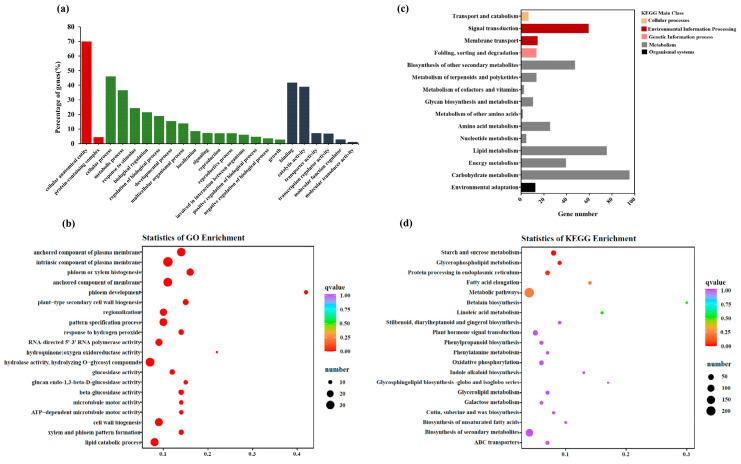
Functional enrichment analysis of DEGs. (**a**) GO classification of all DEGs in Sm64R-grafted eggplant and self-grafted eggplant SG. (**b**) GO enrichment analysis of DEGs. The size of the point indicates the number of enriched DEGs. (**c**) KEGG classification in the main class and subclass of all DEGs. (**d**) KEGG pathway enrichment analysis of DEGs.

These DEGs were separated into three categories and counted as a percentage to analyze their distribution. Finally, 2 biological processes, 15 molecular function, and 6 cellular component categories were selected for analysis. Cellular process and metabolic process were represented highest in the molecular function category. In contrast, only cellular anatomical entities had the highest representation in the cellular component category (Figure 3a). In addition, 20 most significant pathways were identified by GO enrichment analysis of the DEGs. The five most significantly enriched pathways were associated with an intrinsic component of plasma membrane, an anchored component of membrane, hydrolase activity, hydrolysis of O-glycosyl compounds, chemical homeostasis, an anchored component of plasma membrane, and the lipid catabolic process (Figure 3b).

Similar to GO enrichment analysis, DEGs were classified and subjected to KEGG pathway enrichment analysis. In the order of decreasing abundance, the most abundant DEGs were associated with signal transduction, biosynthesis of other secondary metabolites, amino acid metabolism, lipid metabolism, energy metabolism, and carbohydrate metabolism (Figure 3c). The main class of metabolism was the most subclasses with the most enriched genes and were ranked fifth in terms of enrichment significance. Pathway enrichment analysis suggested that the highest enrichment was associated with the biosynthesis of secondary metabolites, plant hormone signal transduction, oxidative phosphorylation, and the activity of these pathways was significantly different between the Sm64R-graft eggplant and self-grafted eggplant SG (Figure 3d).

### 3.6. Correlation Analysis Based on Differentially Expressed Genes and Metabolites

**Figure 4:**
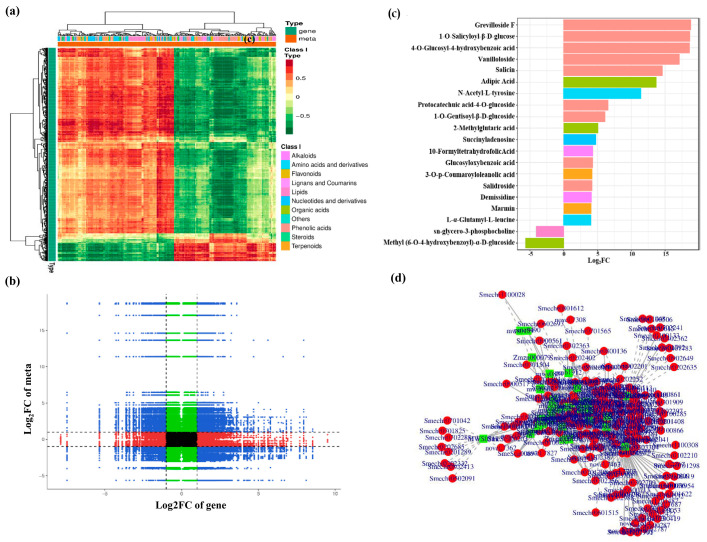
Integrated transcriptomic and metabolomic analyses. (**a**) Heatmap demonstrating the clustering of DEMs and DEGs based on their correlation coefficients. (**b**) The 9-quadrant plot of DEMs and DEGs demonstrates the fold changes in metabolites and genes with Pearson correlation coefficients >0.8 in each group; the plot is divided from left to right and top to bottom into quadrants 1–9 with black dashed lines. (**c**) Classification plot of the top 20 DEMs with the highest change. (**d**) Correlation analysis was performed using Cytoscape and String, with *p* values > 0.001.

A correlation heatmap was drawn to comprehensively classify differentially expressed genes and metabolites. For comparison, metabolites were divided into distinct groups, and the results revealed significant heterogeneity between the Sm64R-graft eggplant and self-grafted eggplant SG (Figure 4a). In particular, the right side of the dendrogram indicated that the DAMs in Sm64R were predominantly phenolic acids, lipids, nucleotides, and derivatives, which were related to the metabolite specificity of Sm64R. A 9-quadrant plot was drawn based on the Pearson correlation coefficients >0.8. Different genes/metabolites are represented by different colors and quadrants as well as by positive or negative regulatory interactions. In contrast to quadrant 5, which showed no significant differentially regulation of DEGs or DEMs, quadrants 1 and 9 showed differently upregulated and downregulated DEGs/DEMs, respectively. Positive regulatory linkages were identified in quadrants 3 and 7, showing that the DEGs and DEMs were interrelated (Figure 4b). The combined KEGG analysis of DEGs and DAMs revealed 25 co-enrichment pathways in the Sm64R-grafted eggplant and self-grafted eggplant SG (Figure S3), among which phenylpropane biosynthesis, ABC transporters, Phenylalanine metabolism, and nucleotide metabolism were the significantly different metabolic pathways between DAMs and DEGs.

As shown in Figure 4c, positively correlated metabolite was upregulated in Sm64R, whereas those negatively correlated metabolites were upregulated in self-grafted SG. The top 20 metabolites were phenolic acids, including grevilloside F, 1-O-salicyloyl-β-D-glucose, 4-O-glucosyl-4-hydroxybenzoic acid, vanilloloside, salicin, protocatechuic acid-4-O-glucoside, 1-O-gentisoyl-β-D-glucoside, glucosyloxybenzoic acid, and salidroside, and significant differences were observed in their expression between Sm64R and self-grafted SG. Furthermore, two amino acids and derivatives (N-acetyl-L-tyrosine, L-α-glutamyl-L-leucine), three organic acids (adipic acid, 2-methylglutaric acid, and methyl (6-O-4-hydroxybenzoyl)-α-D-glucoside), two alkaloids (10-formyltetrahydrofolic acid, demissidine), and one 3-O-p-coumaroyloleanolic acid were upregulated in Sm64R-grafted eggplant compared to self-grafted eggplant SG. More phenolic acids were present in Sm64R-grafted eggplant. The distribution of DAMs revealed variations in fruit composition between the Sm64R-grafted eggplant and self-grafted eggplant SG. Furthermore, the correlation between the DAMs and their related genes was also examined using STRING and Cytoscape. The findings demonstrated that the 23 classes of DEMs (9 classes of nucleotides and derivatives, 5 classes of alkaloids, 4 classes of phenolic acids, 3 classes of organic acids, and 2 classes of amino acids and their derivatives) were highly correlated with DEGs (Figure 4d; Table S3). These DAMs and DEGs were associated with eggplant nutrition.

### 3.7. Correlation Analyses of the Metabolomic and Transcriptomic Data

**Figure 5:**
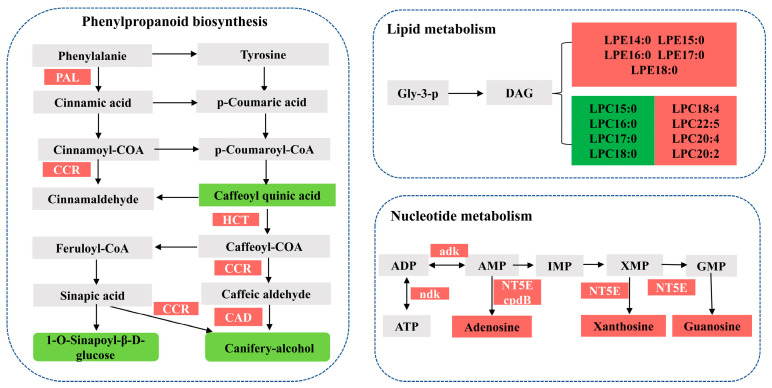
Map of differences in the metabolic pathways between Sm64R-grafted eggplant and self-grafted eggplant SG in phenylpropanoid biosynthesis, lipid metabolism, and nucleotide metabolism. Upregulated and downregulated metabolites/genes are highlighted in red and green, respectively.

The flavours of Sm64R-grafted eggplant and self-grafted eggplant SG were determined based on the differences in nutrients. Based on the metabolome and transcriptome, significant differences were found in metabolites involved in phenylpropanoids, phospholipids, and nucleotide metabolism (Figure 5). In the phenylpropanoid biosynthesis process, caffeoylquinic acid, 1-O-sinapoyl-β-D-glucose, and coniferyl alcohol accumulated in low concentrations in the Sm64R-grafted while grevilloside F, 1-O-salicyloyl-β-D-glucose, vanilloloside, salicin, vanillic acid, and rosmarinic acid were higher in Sm64R, with significant differences in the contents of grevilloside F and salicin (Figure 5). Correspondingly, the Sm64R-grafted eggplant had high expression levels of the enzymes involved in phenylpropanoid biosynthesis process, including PAL, CCR, HCT, and CAD. Statistical analysis of glycerol phospholipids showed a significant increase of LPE and LPC content in Sm64R-grafted eggplant than in self-grafted eggplant SG (Figure 5). Although phospholipase plays a crucial role in the catabolic route of phospholipids and has a marked high expression in Sm64R-grafted eggplant compared to that in self-grafted eggplant SG, we were unable to identify a corresponding synthetic enzyme in this pathway. For the catabolism of purine nucleotides (AMP, IMP, GMP, and XMP), the generation of adenosine, inosine, guanosine, and xanthosine depends on the activity of 5′-nucleotidase. Within nucleotide metabolism, the contents of adenosine, xanthosine, and guanosine were higher in Sm64R than in SG while related enzymes, such as cpdB, NT5E, adk, and ndk, showed higher activity in Sm64R than in SG.

The evidence presented above on differential metabolites and transcripts suggests that there are differences in the nutrient content between Sm64R-graft eggplant and self-grafted eggplant SG and that these differences may be caused by different levels of metabolic enzymes.

## 4. Discussion

Interactions between rootstock and scion are typical in horticultural plants [32], and rootstock sources can have a significant impact on eggplant vigour, yield, and fruit quality. Fruit length and yield, which may be considered an indicator of vigour, were highest in the Sm64R-grafted eggplant compared to self-grafted eggplant SG, demonstrating that rootstock vigour is vital in conferring scion vigour. In our study, greater fruit weight and fruit length were observed in Sm64R-grafted eggplant than in self-grafted eggplant SG. Fruit length has also been observed to be increased in eggplant grafted onto Solanum torvum and watermelon grafted onto pumpkin rootstocks. Increased fruit length has also been observed for eggplant grafted onto S. torvum and in watermelon plants grafted onto pumpkin rootstocks [14,33]. Grafting can influence on product quality of fruit vegetables [34]. In this study, differences in apparent quality were found in the Sm64R-grafted eggplant compared to self-grafted eggplant SG. For example, the presence of TSS was significantly higher in the Sm64R-graft eggplant. Moreover, increased phenolic acid, total amino acid, and vitamin C contents were identified in the Sm64R-graft eggplant. The above physiological traits indicate that the rootstock Sm64R has important value for improving eggplant yield and fruit quality characteristics.

The most significant bioactive components of eggplant are phenolic compounds, which include phenolic acids, anthocyanins, and flavonoids. Research has shown that anthocyanins and lignin are closely related to organ colouring, nutritional quality, and disease and insect resistance in Solanaceae vegetable products. Anthocyanins in eggplant peels are mainly delphinidin-3-rutinoside (D3R) and delphinidin-3-(p-coumarinylrutinoside)-5-glucoside [35]. In total, 11 anthocyanins were identified in eggplant fruits, and 10 of 11 anthocyanins were not significantly different in the Sm64R-grafted eggplant compared to the self-grafted eggplant SG. There was no significant difference in the content of delphinidin-3-rutinoside and delphinidin-3-(p-coumarinylrutinoside)-5-glucoside, suggesting that the rootstock Sm64R does not affect the colour of the eggplant peel. Studies on phenolic acids in eggplants are limited, with phenolic acids receiving less attention than anthocyanins [36,37]. The phenolic compounds detected in the Sm64R-grafted eggplant and self-grafted eggplant SG differed substantially in content, especially grevilloside F, 1-O-salicyloyl-β-D-glucose, vanilloloside, and salicin. In addition, chlorogenic acid-related metabolites are primarily downregulated in the Sm64R-graft eggplant, with significant differences in chlorogenic acid, neochlorogenic acid, isochlorogenic acid A, and isochlorogenic acid B. Chlorogenic acid is the most abundant phenolic metabolite in eggplants, accounting for 80% to 95% of total hydroxy cinnamic acids in the fruit flesh [38,39]. Chlorogenic acid has strong antioxidant capacity and plays an active role in Solanaceae crops regulating fruit development, resistance to biotic or biotic stress, and improving human health [40]. However, studies have shown that astringent compounds mainly include polyphenolic substances, such as chlorogenic acid, neochlorogenic acid, tannins, and catechins [41]. Research has shown that the contents of chlorogenic acid and neochlorogenic acid in fruits are positively correlated with fruit astringency [42,43]. Excessive content of astringent substances can also affect the flavour of the fruit. In-depth studies should be conducted to coordinate flavour and antioxidant capacity nutrition to enrich the edible value of eggplant.

The main primary precursors of ester, ethanol, and aldehyde-volatiles are free fatty acids or those that have been released by lipase activity and subsequently metabolized by oxidative enzymes and/or lipoxygenase [44]. The key to improving fruit flavor is to comprehend the composition of volatile organic compounds. Multiple volatile fatty acids have significant effects on tomato flavor, and lipids are assumed to be their precursors [45]. Twenty and nine differentially accumulated lipids that were upregulated and downregulated, respectively, were identified from the Sm64R-graft eggplant compared to the self-grafted eggplant SG. Intriguingly, several VOCs, including one terpene (robinlin), two ketones (1,7-bis-(4-hydroxyphenyl)-2,4,6-heptatrienone; icariside B2), five esters (5-glucosyloxy-2-hydroxybenzoic acid methyl ester; 3,5-O-dicaffeoylquinic acid methyl ester; ferulic acid methyl ester; 3,4-O-dicaffeoylquinic acid methyl ester; 4,5-O-dicaffeoylquinic acid methyl ester), and one alcohol (eucommiol), were differentially accumulated in the Sm64R-grafted eggplant compared to self-grafted eggplant SG, which may influence the flavour of eggplant (Table S1). However, no valid evidence for a correlation between lipids and VOC was found in eggplants.

Nucleotides are significant flavour enhancers that can significantly improve the flavour and mouthfeel of other compounds, although they have little or no flavour or aroma themselves [46]. In recent years, there has been an increasing focus on food flavours, especially in mushrooms and fish, where metabolic regulatory mechanisms for enhanced umami have now been identified [47,48]. For example, adenosine, xanthosine, and guanosine can synergize with glutamic acid to significantly increase the umami taste. The contents of IMP, UMP, and GMP were relatively high. IMP and GMP both act as flavour enhancers [49]. However, more research is needed in the study of nucleotides in eggplants.

Phenolic acids, nucleotides, lipids, and alkaloids can all contribute to taste differences [50,51]. Nonetheless, the combined effects of these compounds result in distinct flavors. People evaluate eggplant differently due to varieties’ diversity and physiological variances. As a result, various subjective factors, such as the color, flavor, texture, and the nutritional value, are critical to consumer acceptance.

## 5. Conclusions

In this study, a widely cultivated eggplant (*S. melongena* cv. ‘Zheqie No.10’) was selected as the scion and grafted, respectively, onto four rootstocks of TOR (*S. torvum*), Sa (*S. aculeatissimum*), SS (*S. sisymbriifolium*), and Sm64R (*S. melongena* cv. ‘Qiezhen No. 64R’) for phenotypic screening. Physiological and biochemical analysis showed that the rootstock Sm64R has important value for improving eggplant yield and fruit quality characteristics. A systemic investigation of the metabolic difference between the Sm64R-grafted eggplant and self-grafted eggplant SG was performed using LC–MS/MS-based widely targeted metabolomics. Dominant phenolic compounds, higher accumulation of amino acids as well as nucleotides in the Sm64R-graft eggplant might contribute to the taste and flavour difference between the Sm64R-graft eggplant and self-grafted eggplant SG. Moreover, the great difference in the content of secondary metabolites, especially terpenoids and alkaloids, might result in different nutritional characteristics between them. The association study metabolome and transcriptome revealed some key genes in regulating the differences in metabolites between the Sm64R-graft eggplant and self-grafted eggplant SG. Many of these core transcripts might be responsible for encoding vital processes required in differential metabolites. Efforts are needed to conduct further research on molecular biology and characteristic metabolites in eggplants.

## Figures and Tables

**Table 1:** Comparison of fruit length, fruit diameter, fruit weight, hardness, total soluble solids, and titratable acid of eggplant grafted onto different rootstock.

Grafting Combinations	Fruit Length (cm)	Fruit Diameter (cm)	Fruit Weight (g)	Hardness (N)	Total Soluble Solid (Brix^0^)	Titratable Acid (mg/g)
Sa	33.68 ± 0.20 b	2.33 ± 0.03 b	98.4 ± 0.52 b	6.39 ± 0.11 a	14.36 ± 0.30 b	1.58 ± 0.03 c
SS	33.64 ± 0.19 b	2.40 ± 0.02 b	99.2 ± 0.54 b	6.37 ± 0.11 a	13.38 ± 0.17 b	1.76 ± 0.03 c
TOR	38.20 ± 0.16 a	2.58 ± 0.02 a	107.58 ± 0.78 a	6.18 ± 0.18 a	13.42 ± 0.24 b	1.38 ± 0.02 c
Sm64R	39.10 ± 0.17 a	2.61 ± 0.09 a	110.02 ± 0.68 a	6.29 ± 0.15 a	15.78 ± 0.21 a	1.67 ± 0.02 b
SG	33.73 ± 0.22 b	2.40 ± 0.02 b	97.45 ± 0.47 b	6.26 ± 0.09 a	14.00 ± 0.16 b	1.80 ± 0.02 a

Data are the mean ± SE of three replicates. Different lowercase letters indicate significant differences at *p* < 0.05.

**Table 2:** Comparison for the content of fruit flavonoids, anthocyanin, phenolic acid, total sugar, total sugar, titratable acid, and vitamin C of eggplant grafted onto different rootstock.

Grafting Combinations	Flavonoids (ug/g)	Anthocyanin (ug/g)	Phenolic Acid (ug/g)	Total Amino Acid (ug/g)	Total Sugar (mg/g)	Vitamin C (mg/g)
Sa	25.30 ± 1.10 d	2.52 ± 0.23 c	155.46 ± 1.33 b	12.64 ± 0.18 b	23.16 ± 0.47 c	3.61 ± 0.06 d
SS	38.63 ± 1.13 c	6.30 ± 0.34 a	154.05 ± 3.61 b	12.86 ± 0.51 b	18.65 ± 0.29 d	6.12 ± 0.04 ab
TOR	38.46 ± 0.42 c	5.07 ± 0.39 b	139.97 ± 3.73 c	13.82 ± 0.22 a	23.95 ± 0.78 c	5.89 ± 0.20 b
Sm64R	42.60 ± 0.89 b	6.18 ± 0.35 a	207.36 ± 4.53 a	14.85 ± 0.23 a	26.74 ± 0.32 b	6.28 ± 0.12 a
SG	47.01 ± 1.41 a	6.49 ± 0.13 a	153.46 ± 2.18 b	12.31 ± 0.42 b	29.34 ± 1.15 a	4.30 ± 0.12 c

Data are the mean ± SE of three replicates. Different lowercase letters indicate significant differences at *p* < 0.05.

## Data Availability

Data will be made available on request.

## Supplementary Materials

The following supporting information can be downloaded at: https://www.mdpi.com/article/10.3390/foods12163082/s1, Figure S1: KEGG pathway enrichment analysis of DAMs between Sm64R-grafted eggplant and self-grafted eggplant SG; Figure S2: Heatmap of different abundances of phenolic acids between Sm64R-grafted eggplant and self-grafted eggplant SG; Figure S3: Joint KEGG pathway enrichment analysis between DAMs and DEGS. “Meta” represents KEGG pathway enriched by DAMs, and “Gene” represents KEGG pathway enriched by DEGs; Table S1: List of all the differential metabolites identified between Sm64R-grafted eggplant and self-grafted eggplant SG; Table S2: List of all the differentially expressed genes identified between Sm64R-grafted eggplant and self-grafted eggplant SG; Table S3: List of differential metabolites and genes with Pearson correlation coefficients > 0.8.
