# Supplementary figures and images for: Metabolomic and Transcriptomic Analyses Reveal the Effects of Grafting on Nutritional Properties in Eggplant
Source: Foods. 2023 Aug 17;12(16):3082. doi: 10.3390/foods12163082 (PMC10453275; doi:10.3390/foods12163082)

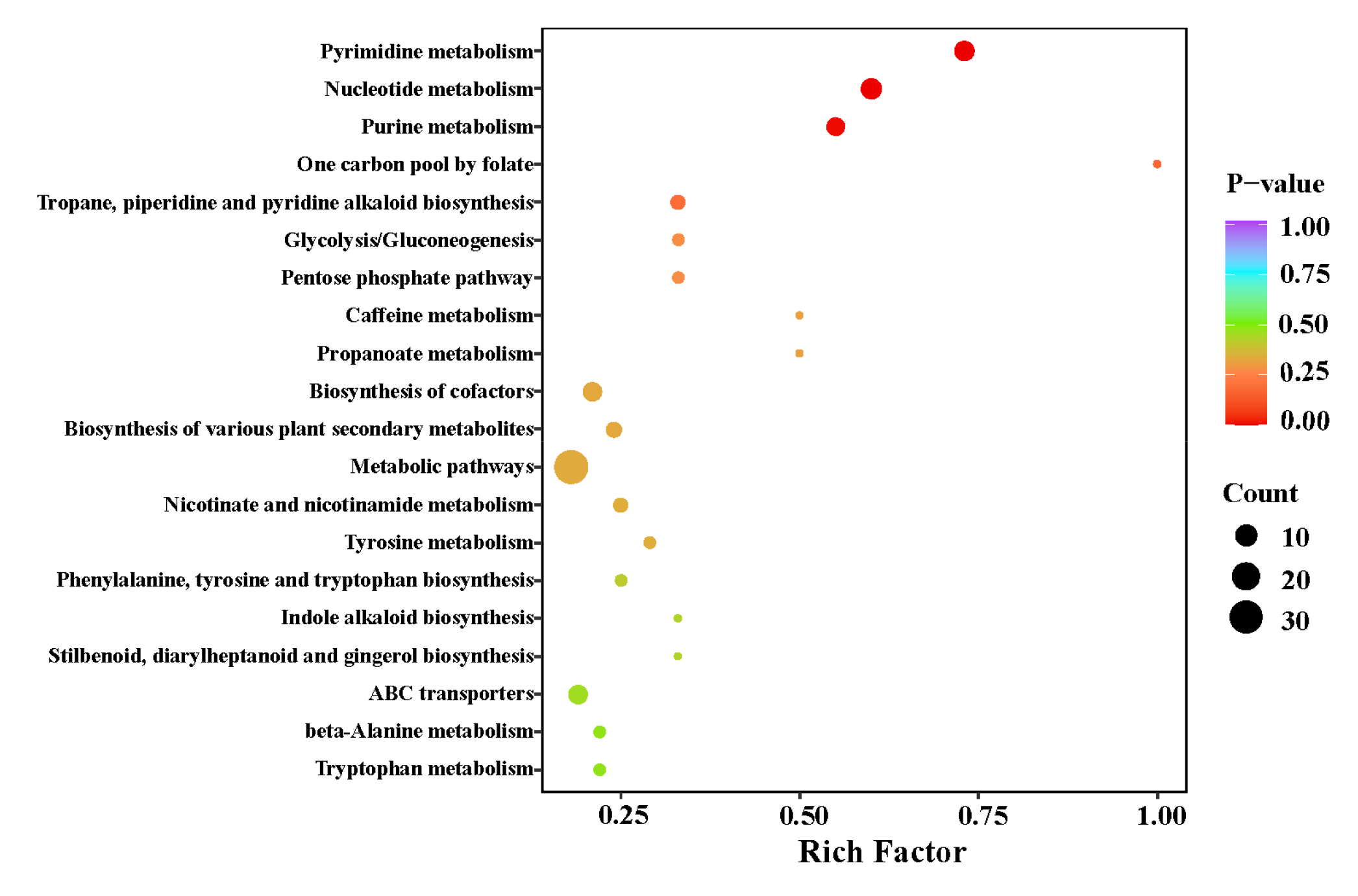

Supplement: Supplementary file 1 [file foods-12-03082-s001.zip › Supplementary material/Figure.S1.tif]

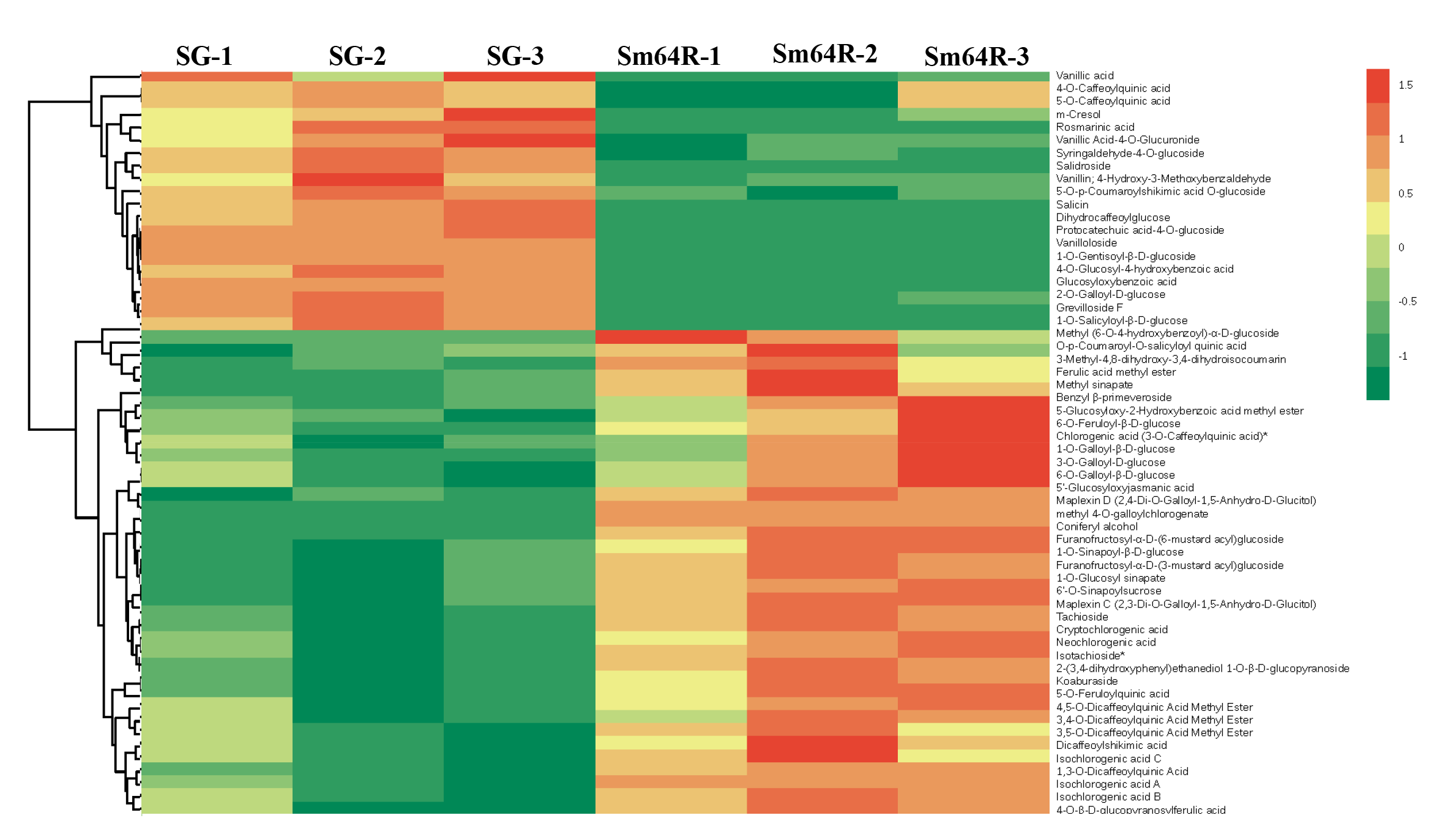

Supplement: Supplementary file 1 [file foods-12-03082-s001.zip › Supplementary material/Figure.S2.tif]

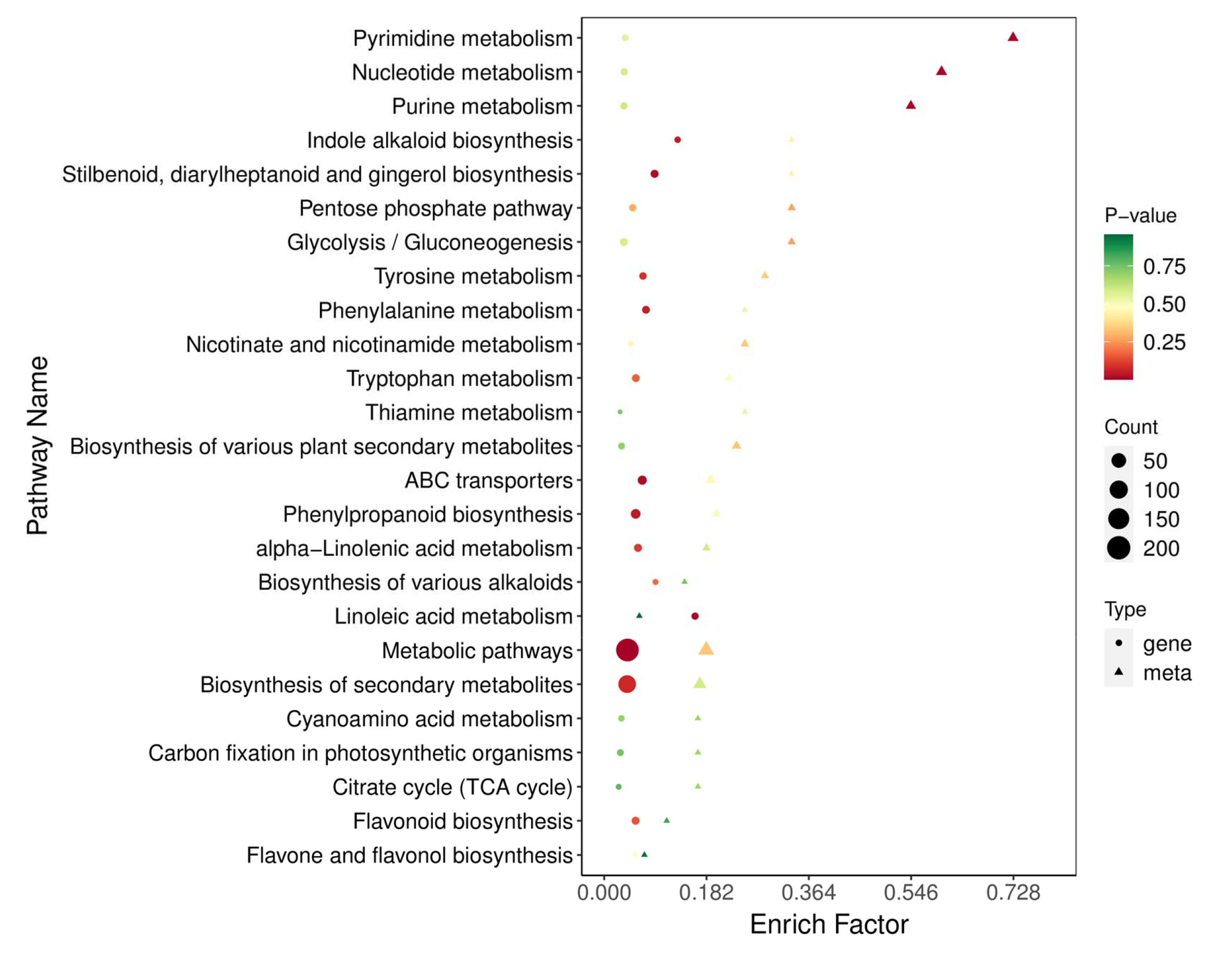

Supplement: Supplementary file 1 [file foods-12-03082-s001.zip › Supplementary material/Figure.S3.tif]
